# Lipopolysaccharides from Different *Burkholderia* Species with Different Lipid A Structures Induce Toll-Like Receptor 4 Activation and React with Melioidosis Patient Sera

**DOI:** 10.1128/IAI.00692-19

**Published:** 2019-11-18

**Authors:** Sineenart Sengyee, Sung Hwan Yoon, T. Eoin West, Robert K. Ernst, Narisara Chantratita

**Affiliations:** a Department of Microbiology and Immunology, Faculty of Tropical Medicine, Mahidol University, Bangkok, Thailand; b Department of Microbial Pathogenesis, University of Maryland, Baltimore, Maryland, USA; c Division of Pulmonary and Critical Care Medicine, Harborview Medical Center, University of Washington, Seattle, Washington, USA; d International Respiratory and Severe Illness Center, University of Washington, Seattle, Washington, USA; e Mahidol-Oxford Tropical Medicine Research Unit, Faculty of Tropical Medicine, Mahidol University, Bangkok, Thailand; University of California—San Diego School of Medicine

**Keywords:** *Burkholderia*, lipopolysaccharide, O-polysaccharide, TLR4, melioidosis, antibody, bacterial infection, LPS structure, diagnostics, innate immunity, serology

## Abstract

Lipopolysaccharides (LPSs) of Gram-negative bacteria comprise lipid A, core, and O-polysaccharide (OPS) components. Studies have demonstrated that LPSs isolated from the pathogenic species Burkholderia pseudomallei and Burkholderia mallei and from less-pathogenic species, such as Burkholderia thailandensis, are potent immune stimulators. The LPS structure of B. pseudomallei, the causative agent of melioidosis, is highly conserved in isolates from Thailand; however, the LPSs isolated from other, related species have not been characterized to enable understanding of their immune recognition and antigenicities. Here, we describe the structural and immunological characteristics of the LPSs isolated from eight *Burkholderia* species and compare those for B. pseudomallei to those for the other seven species. Matrix-assisted laser desorption ionization–time of flight mass spectrometry (MALDI-TOF MS), gas chromatography (GC), SDS-PAGE, Toll-like receptor 4 (TLR4) stimulation, and immunoblot analysis were performed on these *Burkholderia* species. MALDI-TOF profiles demonstrated that *Burkholderia* lipid A contains predominantly penta-acylated species modified with 4-amino-4-deoxy-arabinose residues at both terminal phosphate groups. The lipid A could be differentiated based on mass differences at *m/z* 1,511, 1,642, 1,773, and 1,926 and on fatty acid composition. LPSs of all species induced TLR4-dependent NF-κB responses; however, while SDS-PAGE analysis showed similar LPS ladder patterns for B. pseudomallei, B. thailandensis, and B. mallei, these patterns differed from those of other *Burkholderia* species. Interestingly, immunoblot analysis demonstrated that melioidosis patient sera cross-reacted with OPSs of other *Burkholderia* species. These findings can be used to better understand the characteristics of LPS in *Burkholderia* species, and they have implications for serological diagnostics based on the detection of antibodies to OPS.

## Abstract (precis)

Lipopolysaccharides (LPSs) of Gram-negative bacteria comprise lipid A, core, and O-polysaccharide (OPS) components. Studies have demonstrated that LPSs isolated from the pathogenic species Burkholderia pseudomallei and Burkholderia mallei and from less-pathogenic species, such as Burkholderia thailandensis, are potent immune stimulators.

## INTRODUCTION

Members of the genus *Burkholderia* are Gram-negative rod-shaped bacteria comprising both pathogenic and nonpathogenic species. Burkholderia pseudomallei and B. mallei are primary pathogens of animals and humans that are categorized as Tier 1 select agents because of their potential use for biological terrorism (1). B. pseudomallei is the causative agent of melioidosis, an infection for which the estimated global burden is 165,000 cases and the predicted mortality is 89,000 deaths per year (2). B. mallei is the causative agent of glanders, which is responsible primarily for disease in animals and occasionally in humans (3, 4). Other members of the genus, including the less-pathogenic B. thailandensis and B. oklahomensis species, are closely related to B. pseudomallei and B. mallei (5). In addition to the B. pseudomallei group, there is a group currently comprising 20 related bacterial species, referred to as the Burkholderia cepacia complex (Bcc), that have emerged as opportunistic pathogens capable of causing severe infections in cystic fibrosis (CF) and immunocompromised patients (6). All Bcc species have been isolated from the natural environment, including soil samples or the rhizospheres of various plants. B. cepacia, B. multivorans, B. vietnamiensis, and B. ubonensis are representative members of the Bcc group (6, 7). Like melioidosis and glanders patients, patients infected with Bcc species demonstrate highly variable clinical presentations and outcomes. In some cases, patients infected with Bcc species experience a rapid decline of lung function, leading to a fatal necrotizing pneumonia (8–10). The sites of *Burkholderia* species infection mostly involve the lungs, bloodstream, skin, and soft tissue. The course of infection may differ depending on the bacterial strains, virulence factors, and host determinants. B. pseudomallei and B. mallei are highly virulent, in contrast to B. thailandensis and B. oklahomensis, which have been described as species closely related to B. pseudomallei. B. thailandensis and B. oklahomensis rarely cause human (5, 11, 12) or animal (13) infections. B. multivorans and B. cenocepacia are the commonest species within the Bcc that cause infection in CF patients (14). Patients may be coinfected, at least transiently, with more than one Bcc strain (15–17). Therefore, it is relevant to evaluate bacterial membrane differences that may relate to these clinical observations.

Lipopolysaccharide (LPS) is the major component of the outer membrane of Gram-negative bacteria (18). Bacterial LPS typically consists of lipid A, a core oligosaccharide, and a distal O-polysaccharide (OPS). Lipid A is the endotoxic portion of LPS that is crucial in eliciting mammalian innate immunity. It represents the pathogen-associated molecular pattern (PAMP) that is recognized by the Toll-like receptor 4 (TLR4)–MD2 receptor complex. LPS-TLR4 ligation initiates NF-ĸB activation and a subsequent inflammatory response leading to the expression of cytokines, chemokines, prostaglandins, and reactive oxygen species, which manifests as acute inflammation during infection (19, 20). The immune responses to LPSs isolated from different Gram-negative bacteria differ strongly with the primary structures of the lipid A and OPS molecules that interact with the immune cells (18).

Antibodies in serum samples from patients with B. pseudomallei infection (melioidosis) cross-react with the OPSs of B. mallei and B. thailandensis (21, 22). This finding suggests strong antigenic relatedness among the OPSs of this group of organisms. Antigenic cross-reactivity with other *Burkholderia* species, including B. oklahomensis, B. ubonensis, and B. thailandensis-like species, has been reported previously for a melioidosis patient from Australia (23). However, no study has reported antibody cross-reactivity with the OPSs isolated from B. cepacia, B. multivorans, B. vietnamiensis, or B. ubonensis. Our recent study demonstrated that the LPS of B. pseudomallei can induce TLR4-dependent NF-κB activation and that the lipid A structures of 171 clinical and environmental isolates in Thailand are highly conserved, represented by penta- and tetra-acylated, bisphosphorylated disaccharide backbones modified with 4-amino-4-deoxy-arabinose (Ara4N) (24). Other reports suggest that B. thailandensis and B. mallei lipid A species are composed of the same backbone structure with potential differences in fatty acid composition (25, 26). Bcc lipid A species, including those of B. cepacia, B. multivorans, and B. vietnamiensis, also contain a penta-acylated, bisphosphorylated disaccharide backbone modified with Ara4N (7, 27–30). These lipid A species have been shown to be potent immunostimulatory molecules (7, 25–29) and may be linked to successful infection by these *Burkholderia* species and to human diseases.

Therefore, there is a need to understand the role of TLR4-mediated immune signaling by different *Burkholderia* lipid A species in the recognition of LPS by the host innate immune system. Several published papers have characterized *Burkholderia* lipid A species using various methods, making it difficult to compare immunological responses correlating with specific structural features of lipid A (25–27, 31). Here, we used matrix-assisted laser desorption ionization–time of flight mass spectrometry (MALDI-TOF MS) followed by gas chromatography (GC) to compare the lipid A structures of seven genetically related *Burkholderia* species with the lipid A structure of B. pseudomallei. Since the lipid A structures of different species may correlate with different levels of innate immune activation through TLR4, we thus compared TLR4-dependent NF-κB activation by these LPSs using a HEK-Blue human TLR4 (hTLR4) reporter cell line. In addition, we used SDS-PAGE to evaluate LPS patterns, and we used immunoblotting to observe the antigenic cross-reactivities of these LPSs with sera from several melioidosis patients and healthy donors. The cross-reactivities of antibodies to LPSs have important implications for serological diagnostics based on antibody detection of LPS.

## RESULTS

### Structural analysis of *Burkholderia* species.

**FIG 1:**
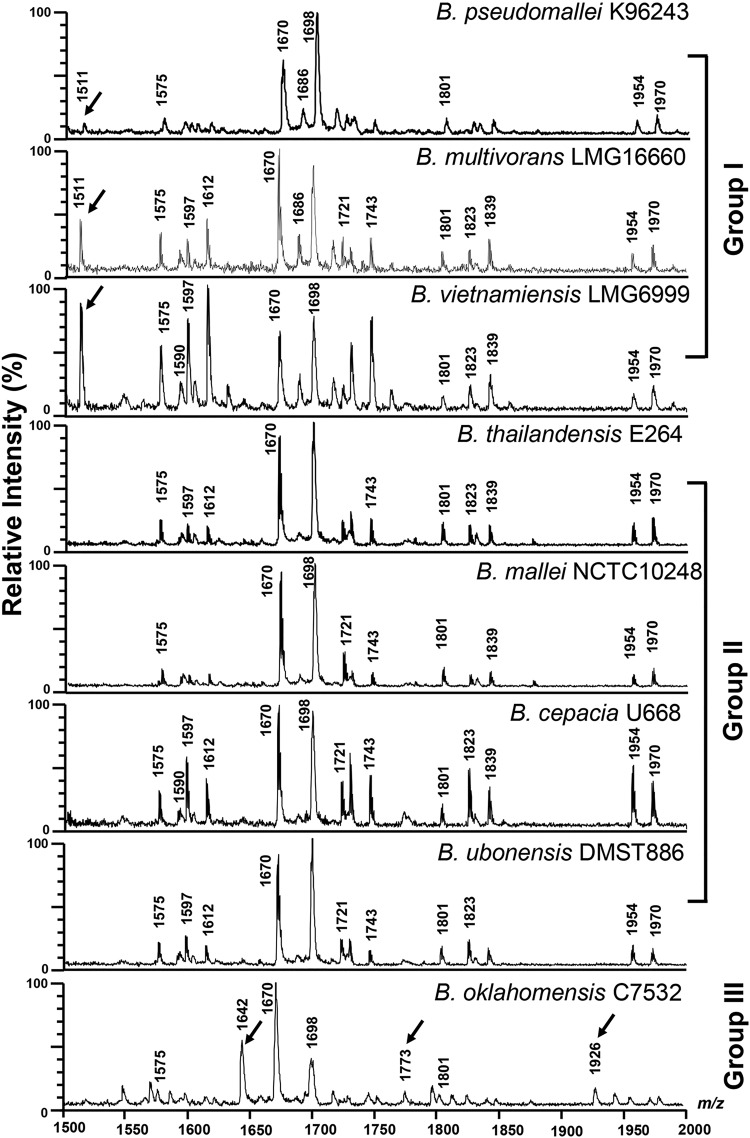
MALDI-TOF spectra of lipid A from representative strains of eight different *Burkholderia* species.

The lipid A structures of all 19 strains of the eight *Burkholderia* species studied (2 B. pseudomallei strains, 2 B. thailandensis strains, 4 B. mallei strains, 5 B. cepacia strains, 1 B. multivorans strain, 3 B. oklahomensis strains, 1 B. vietnamiensis strain, and 1 B. ubonensis strain) were initially characterized using MALDI-TOF MS in the negative-ion mode. Different strains of the same species showed similar spectra between *m/z* 1,500 and *m/z* 2,000. These spectra were divided into three groups with different ion masses at *m/z* 1,511, 1,642, 1,773, and 1,926 (proposed structures described below). A representative spectrum for each *Burkholderia* species is shown in Fig. 1. The representative strains are B. pseudomallei K96243, B. multivorans LMG16660, B. vietnamiensis LMG6999, B. thailandensis E264, B. mallei NCTC10248, B. cepacia U668, B. ubonensis DMST886, and B. oklahomensis C7532.

**FIG 2:**
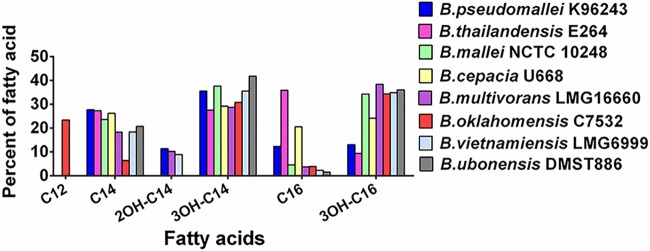
Fatty acid components in lipopolysaccharides of representative strains of eight *Burkholderia* species.

**FIG 3:**
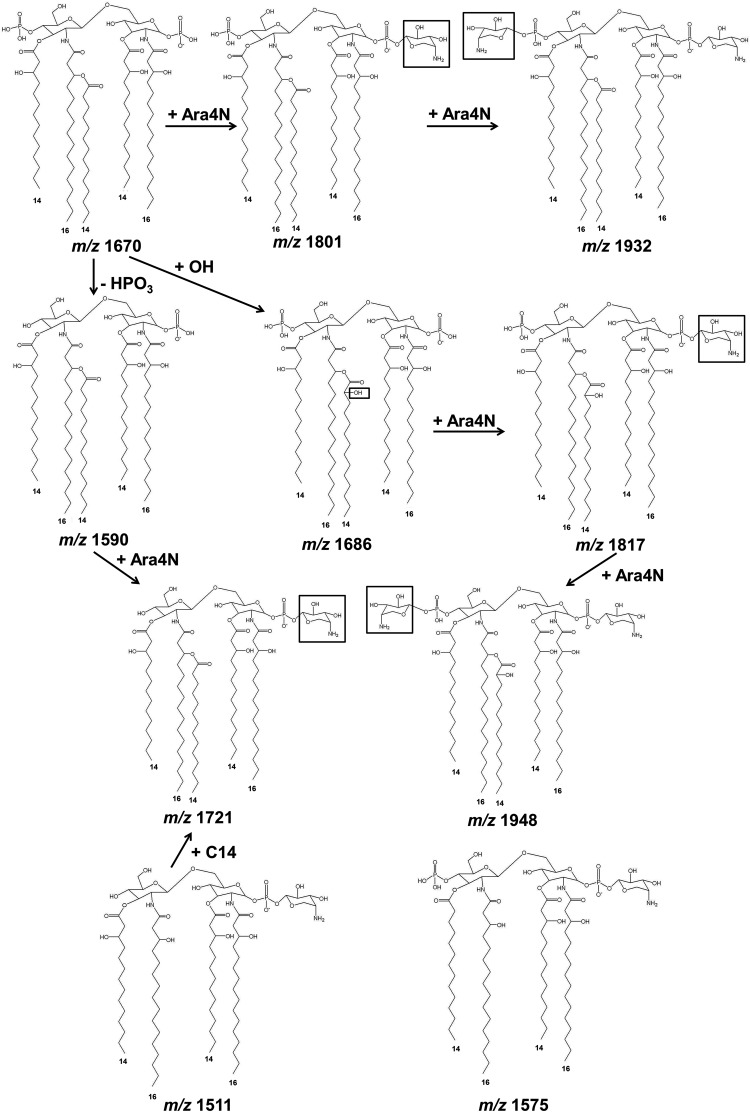
Lipid A structures of B. pseudomallei K96243, B. multivorans LMG16660, and B. vietnamiensis LMG6999.

The first group, composed of B. pseudomallei K96243, B. multivorans LMG16660, and B. vietnamiensis LMG6999, demonstrated similar lipid A spectra with ions at *m/z* 1,511, 1,575, 1,590, 1,597, 1,612, 1,670, 1,686, 1,698, 1,721, 1,743, 1,801, 1,823, 1,839, 1,954, and 1,970 (Fig. 1 and Table 1). The ion at *m/z* 1,511 represents a tetra-acylated, monophosphorylated GlcN disaccharide backbone possessing two 3-hydroxytetradecanoic acid [C_14:0_(3-OH)] residues in ester linkage and two 3-hydroxyhexadecanoic acid [C_16:0_(3-OH)] residues in amide linkage with one phosphorylated residue and one 4-amino-4-deoxy-arabinose (Ara4N) residue attached to the phosphate group. The ion at *m/z* 1,575 represents a tetra-acylated, bisphosphorylated GlcN disaccharide backbone possessing one tetradecanoic acid (C_14:0_) residue and one C_14:0_(3-OH) residue in ester linkage and two C_16:0_(3-OH) residues in amide linkage, with one Ara4N residue attached to the phosphate group. The ion at *m/z* 1,590 represents a penta-acylated, monophosphorylated GlcN disaccharide backbone possessing one C_14:0_, two C_14:0_(3-OH), and two C_16:0_(3-OH) residues with one phosphorylated residue. The ions at *m/z* 1,597 and 1,612 represent the ions at *m/z* 1,575 and 1,590, respectively, modified with a sodium substitution (Δ*m/z +*22). The ion at *m/z* 1,670 represents a penta-acylated, bisphosphorylated GlcN disaccharide backbone possessing one C_14:0_ residue, two C_14:0_(3-OH) residues, and two C_16:0_(3-OH) residues. The ion at *m/z* 1,686 represents the ion at *m/z* 1,670 with the substitution of fatty acid C_14:0_(2-OH) for C_14:0_. The ion at *m/z* 1,721 represents the ion at *m/z* 1,590 modified with one Ara4N residue attached to the phosphate group (Δ*m/z* +131). The ion at *m/z* 1,743 represents the ion at *m/z* 1,721 modified with a sodium substitution (Δ*m/z* +22). The ion at *m/z* 1,801 is modified with an Ara4N residue attached to the phosphate group of the ion at *m/z* 1,670. The ion at *m/z* 1,823 represents the ion at *m/z* 1,801 modified with a sodium substitution (Δ*m/z* +22). The ion at *m/z* 1,839 represents the ion at *m/z* 1,686 with a sodium substitution (Δ*m/z* +22) and one Ara4N residue (Δ*m/z* +131) attached to the phosphate group. The ion at *m/z* 1,954 was modified by a sodium substitution (Δ*m/z* +22) and one additional Ara4N residue (Δ*m/z* +131) attached to the phosphate group of the ion at *m/z* 1,801 (24). The ion at *m/z* 1,970 represents the ion at *m/z* 1,686 modified with a sodium substitution (Δ*m/z* +22) and two Ara4N residues (Δ*m/z* +262) attached to the phosphate group (Fig. 1 and Table 1). GC analysis confirmed that the fatty acid composition of bacteria in this group included C_14:0_, C_14:0_(2-OH), C_14:0_(3-OH), C_16:0_, and C_16:0_(3-OH) (Fig. 2). Our data suggest that the lipid A species of the first group were predominantly bisphosphorylated and penta-acylated [with a combination of C_14:0_(2-OH), C_16:0_(3-OH) or C_16:0_, and C_14:0_(3-OH) or C_14:0_] (Fig. 3).

**TABLE 1:** Lipid A species characterized by negative-ion MALDI-TOF MS and predicted structures of the fatty acid, phosphate, and carbohydrate substituents of eight different *Burkholderia* species lipid A backbones

Lipid A species	Observed ion (*m/z*)	Theoretical monoisotopic ion (*m/z*)	Acyl substitution	Predicted structure
1	1,511	1,511.0	Tetra	2 × C_14:0_(3-OH), 2 × C_16:0_(3-OH), P, Ara4N
2	1,575	1,575.0	Tetra	C_14:0_, C_14:0_(3-OH), 2 × C_16:0_(3-OH), 2 × P, Ara4N
3	1,590	1,590.1	Penta	C_14:0_, 2 × C_14:0_(3-OH), 2 × C_16:0_(3-OH), P
4	1,642^*a*^	1,642.0	Penta	C_12_, 2 × C_14:0_(3-OH), 2 × C_16:0_(3-OH), 2 × P
5	1,670	1,670.1	Penta	C_14:0_, 2 × C_14:0_(3-OH), 2 × C_16:0_(3-OH), 2 × P
6	1,686	1,686.1	Penta	C_14:0_(2-OH), 2 × C_14:0_(3-OH), 2 × C_16:0_(3-OH), 2 × P
				C_14:0_(3-OH), 2 × C_14:0_(3-OH), 2 × C_16:0_(3-OH), 2 × P^*b*^
7	1,721	1,721.2	Penta	C_14:0_, 2 × C_14:0_(3-OH), 2 × C_16:0_(3-OH), P, Ara4N
8	1,773^*a*^	1,773.0	Penta	C_12_, 2 × C_14:0_(3-OH), 2 × C_16:0_(3-OH), 2 × P, Ara4N
9	1,801	1,801.1	Penta	C_14:0_, 2 × C_14:0_(3-OH), 2 × C_16:0_(3-OH), 2 × P, Ara4N
10	1,839^*c*^	1,817.2	Penta	C_14:0_(2-OH), 2 × C_14:0_(3-OH), 2 × C_16:0_(3-OH), 2 × P, Ara4N
				C_14:0_(3-OH), 2 ×C_14:0_(3-OH), 2 × C_16:0_(3-OH), 2 × P, Ara4N^*b*^
11	1,926^*a*^^,^^*c*^	1,904.0	Penta	C_12_, 2 × C_14:0_(3-OH), 2 × C_16:0_(3-OH), 2 × P, 2 × Ara4N
12	1,954^*c*^	1,932.2	Penta	C_14:0_, 2 × C_14:0_(3-OH), 2 × C_16:0_(3-OH), 2 × P, 2 × Ara4N
13	1,970^*c*^	1,948.2	Penta	C_14:0_(2-OH), 2 × C_14:0_(3-OH), 2 × C_16:0_(3-OH), 2 × P, 2 × Ara4N
				C_14:0_(3-OH), 2 × C_14:0_(3-OH), 2 × C_16:0_(3-OH), 2 × P, 2 × Ara4N^*b*^

a Lipid A species found only in B. oklahomensis C7532.

b Predicted structure for B. thailandensis E264, B. mallei NCTC10248, B. cepacia U668, and B. ubonensis DMST886.

c Sodium substitution (Δ*m/z* +22).

**FIG 4:**
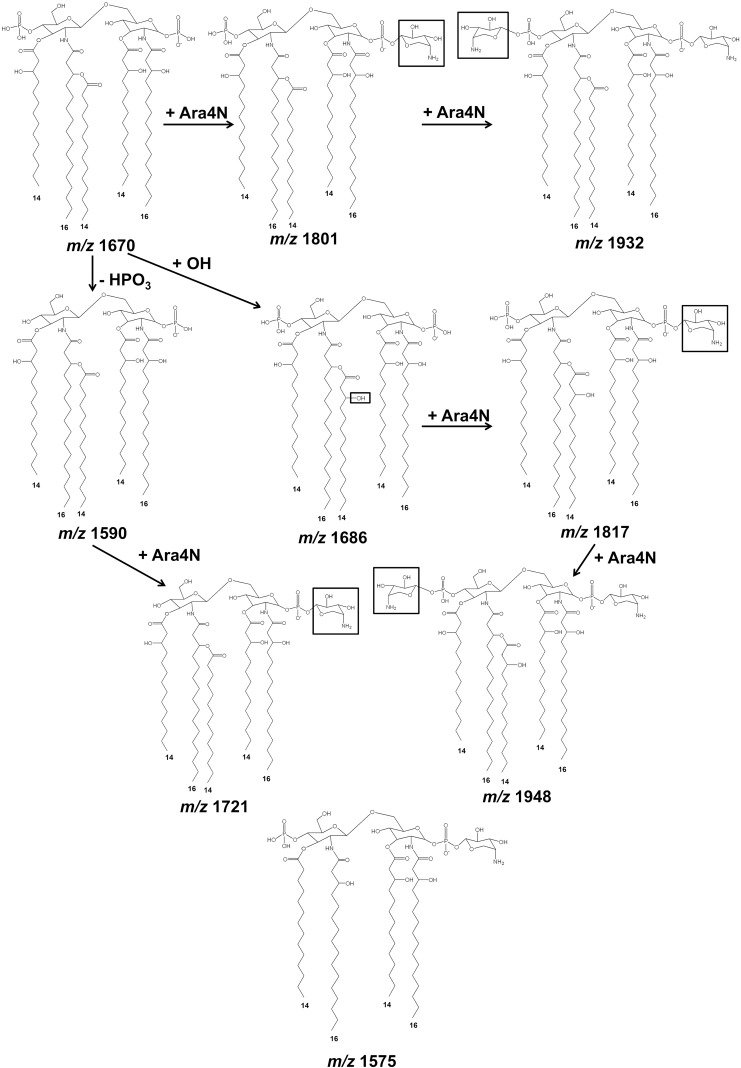
Lipid A structures of B. thailandensis E264, B. mallei NCTC10248, B. cepacia U668, and B. ubonensis DMST886.

The second group included lipid A species of B. thailandensis E264, B. mallei NCTC10248, B. cepacia U668, and B. ubonensis DMST886. The lipid A spectra were observed at *m/z* 1,575, 1,590, 1,597, 1,612, 1,670, 1,686, 1,698, 1,721, 1,743, 1,801, 1,823, 1,839, 1,954, and 1,970 (Fig. 1 and Table 1). The spectra were similar to those of the first group except for the absence of an ion at *m/z* 1,511 (Fig. 1). GC analysis revealed the presence of C_14:0_, C_14:0_(3-OH), C_16:0_, and C_16:0_(3-OH) fatty acids (Fig. 2). However, in contrast to the first group, we did not observe the C_14:0_(2-OH) fatty acid in the second group. This can lead to lipid A structures different from those of the first group at *m/z* 1,686, 1,839, and 1,970 (Fig. 4 and Table 1). Based on overall chemical composition, the data predicted that the lipid A species of the second group were predominantly bisphosphorylated and penta-acylated [with a combination of C_16:0_(3-OH) or C_16:0_ and C_14:0_(3-OH) or C_14:0_] (Fig. 4).

**FIG 5:**
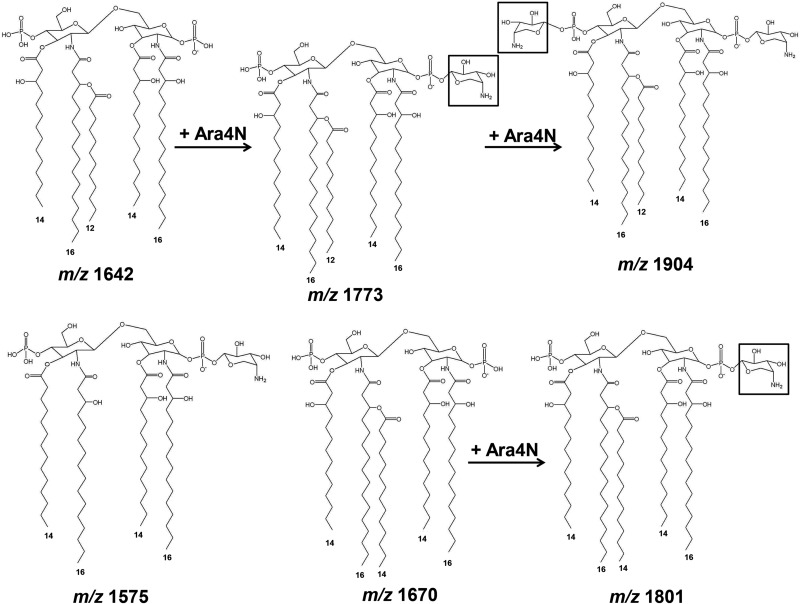
Lipid A structures of B. oklahomensis C7532.

The third group was represented only by B. oklahomensis C7532, which demonstrated a lipid A profile distinct from those of other *Burkholderia* species, with a complex pattern of ions at *m/z* 1,642, 1,773, and 1,926 (Fig. 1). The ion at *m/z* 1,642 represents a penta-acylated, bisphosphorylated GlcN disaccharide backbone possessing one dodecanoic acid (C_12_) residue, two C_14:0_(3-OH) residues in ester linkage, and two C_16:0_(3-OH) residues in amide linkage. The ion at *m/z* 1,773 represents the ion at *m/z* 1,642 modified with one Ara4N residue attached to the phosphate group (Δ*m/z* +131). The ion at *m/z* 1,926 represents the ion at *m/z* 1,773, with a penta-acylated, bisphosphorylated GlcN disaccharide backbone, modified with a sodium substitution (Δ*m/z* +22) and with one Ara4N residue (Δ*m/z* +131) attached to the phosphate group. GC analysis demonstrated the presence of C_12_, C_14:0_, C_14:0_(3-OH), C_16:0_, and C_16:0_(3-OH) fatty acids (Fig. 2). The predicted structures of fatty acid, phosphate, and carbohydrate substituents of B. oklahomensis C7532 lipid A were bisphosphorylated and penta-acylated [with a combination of C_14_(3-OH), C_16:0_(3-OH) or C_16:0_, and C_12:0_] (Fig. 5).

The ion at *m/z* 1,698 that was observed in the lipid A spectra of eight representative *Burkholderia* species (Fig. 1) was not further investigated in this study. Detailed structural analysis of this species has been reported using electrospray ionization (ESI) tandem MS and GC (24), and *m/z* 1,698 was not observed by ESI. Our MALDI-TOF instrument does not have a tandem MS capability to analyze this peak.

### NF-κB activation by lipopolysaccharides from different *Burkholderia* species.

**FIG 6:**
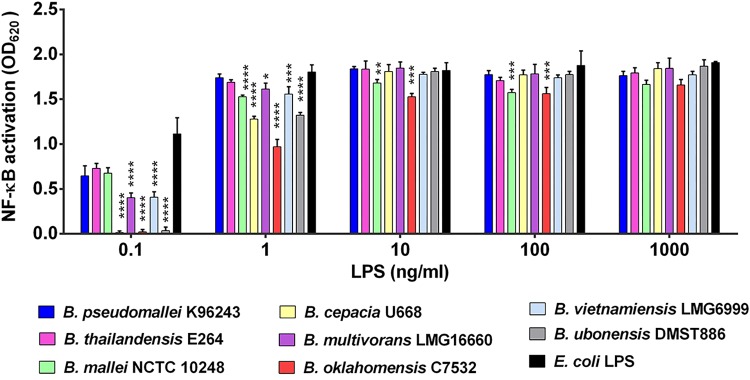
NF-κB activation in HEK-Blue hTLR4 cells by LPSs of representative strains of eight different *Burkholderia* species. The HEK-Blue hTLR4 cells were stimulated with LPSs from eight *Burkholderia* species at 0.1, 1, 10, 100, and 1,000 ng/ml at 37°C under 5% CO_2_ for 24 h. NF-κB activation was determined in the supernatants by use of a SEAP reporter assay. Means ± SD of the results of two independent experiments are shown. The ultrapure TLR4 ligand Escherichia coli O111:B4 LPS (Sigma-Aldrich, MO, USA) was used as a positive control. The mean values for NF-κB activation by the LPSs from different *Burkholderia* species were compared with that for B. pseudomallei LPS. *, *P* < 0.05; **, *P* < 0.01; ***, *P* < 0.001; ****, *P* < 0.0001.

Since lipid A is the endotoxic component of LPS, we tested individual LPSs isolated from representative strains of *Burkholderia* species for the ability to differentially activate the host TLR4–MD2 complex. By use of a reporter cell line that measures NF-κB-driven innate immune activation (HEK-Blue hTLR4), LPSs isolated from the eight *Burkholderia* species were evaluated for the ability to induce a proinflammatory response. NF-κB activation at 24 h after stimulation using a wide range of LPS concentrations (0.1, 1, 10, 100, and 1,000 ng/ml) showed that all eight *Burkholderia* species (B. pseudomallei K96243, B. thailandensis E264, B. mallei NCTC10248, B. cepacia U668, B. multivorans LMG16660, B. oklahomensis C7532, B. vietnamiensis LMG6999, and B. ubonensis DMST886) were able to induce detectable NF-κB activation (Fig. 6). The lowest concentration at which we could detect NF-κB activation when cells were stimulated with the LPSs from B. cepacia, B. oklahomensis, and B. ubonensis was 0.1 ng/ml.

Compared to the level of NF-κB activation after stimulation with B. pseudomallei LPS, we observed lower activation in cells stimulated with LPSs from B. cepacia, B. multivorans, B. oklahomensis, B. vietnamiensis, and B. ubonensis at a concentration of 0.1 ng/ml and in cells stimulated with LPSs from B. mallei, B. cepacia, B. multivorans, B. oklahomensis, B. vietnamiensis, and B. ubonensis at 1 ng/ml (Fig. 6). At concentrations of 10 and 100 ng/ml LPS, we observed lower NF-κB activation in cells stimulated with B. mallei and B. oklahomensis. Finally, we detected similar levels of NF-κB activation after stimulation with LPSs from all *Burkholderia* species at a concentration of 1,000 ng/ml.

### Lipopolysaccharide patterns of different *Burkholderia* species.

**FIG 7:**
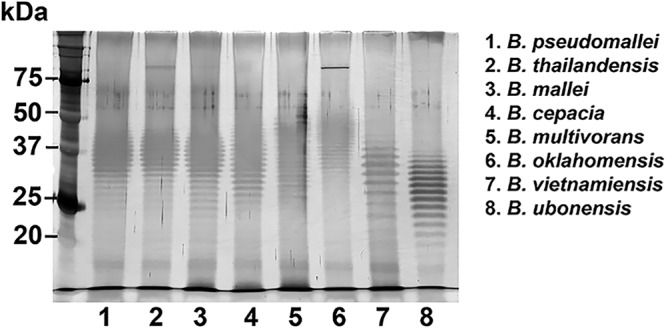
Lipopolysaccharide banding patterns of representative strains of eight *Burkholderia* species on a silver-stained SDS-PAGE gel. Shown are lipopolysaccharide antigens expressed by B. pseudomallei K96243 (lane 1), B. thailandensis E264 (lane 2), B. mallei NCTC10248 (lane 3), B. cepacia U668 (lane 4), B. multivorans LMG16660 (lane 5), B. oklahomensis C7532 (lane 6), B. vietnamiensis LMG6999 (lane 7), and B. ubonensis DMST886 (lane 8).

Silver-stained profiles of LPSs from representative strains of all *Burkholderia* species showed ladder patterns after SDS-PAGE gel analysis. B. pseudomallei K96243, B. thailandensis E264, and B. mallei NCTC10248 showed similar repeating bands with uniform spacing of OPS. B. cepacia U668, B. multivorans LMG16660, B. oklahomensis C7532, B. vietnamiensis LMG6999, and B. ubonensis DMST886 demonstrated different OPS banding patterns (Fig. 7). Therefore, we categorized the LPSs of *Burkholderia* species into two groups based on SDS-PAGE profiles: group I, comprising B. pseudomallei, B. mallei, and B. thailandensis, demonstrated an LPS type A ladder, and group II, comprising B. cepacia, B. multivorans, B. oklahomensis, B. vietnamiensis, and B. ubonensis, demonstrated LPS ladders different from those of group I and from each other.

### Antibody reactions of sera from melioidosis patients and healthy donors with lipopolysaccharides of different *Burkholderia* species.

**FIG 8:**
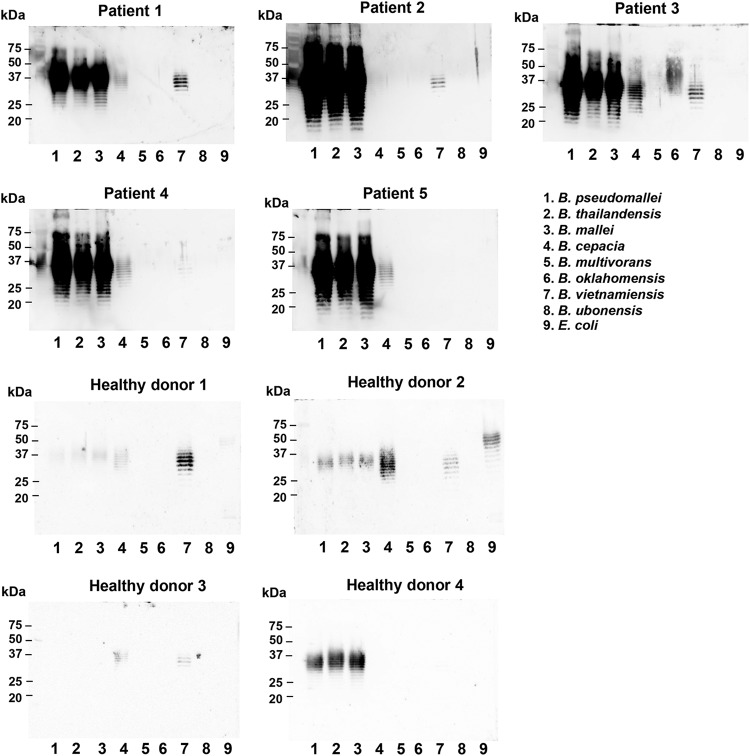
Immunoreactivities of lipopolysaccharides with sera from melioidosis patients and healthy donors. Immunoblot analysis was performed on LPSs from representative strains of eight *Burkholderia* species probed with sera from five melioidosis patients infected with B. pseudomallei type A LPS and four healthy donors at a dilution of 1:1,000. Shown are lipopolysaccharide antigens expressed by B. pseudomallei K96243 (lane 1), B. thailandensis E264 (lane 2), B. mallei NCTC10248 (lane 3), B. cepacia U668 (lane 4), B. multivorans LMG16660 (lane 5), B. oklahomensis C7532 (lane 6), B. vietnamiensis LMG6999 (lane 7), B. ubonensis DMST886 (lane 8), and E. coli (lane 9).

To evaluate the antigenic cross-reactivity between LPSs of B. pseudomallei K96243, B. thailandensis E264, B. mallei NCTC10248, B. cepacia U668, B. multivorans LMG16660, B. oklahomensis C7532, B. vietnamiensis LMG6999, and B. ubonensis DMST886, we performed Western blot analysis using serum samples from five melioidosis patients previously identified as having B. pseudomallei infections (LPS serotype A), in addition to sera from four healthy donors (Fig. 8 and Table 2). LPSs from B. pseudomallei, B. thailandensis, and B. mallei reacted strongly with sera from patients with melioidosis. However, LPSs from other *Burkholderia* species, including B. cepacia, B. vietnamiensis, and B. oklahomensis, demonstrated variable reactions with sera from individual melioidosis patients (Fig. 8 and Table 2). Interestingly, serum samples from healthy donors from northeast Thailand showed positive reactions with LPSs from two to six species (B. pseudomallei, B. thailandensis, B. mallei, B. cepacia, B. vietnamiensis, and Escherichia coli), suggesting that these serological cross-reactivities may impact on diagnostics based on antibody detection of OPS.

**TABLE 2:** Immunoreactivities of lipopolysaccharides of representative strains of eight closely related *Burkholderia* species and E. coli with serum samples from five individual melioidosis patients and four healthy donors

No.	Bacterial LPS	Reaction^*a*^ with serum sample from:								
		Patient					Healthy donor			
		1	2	3	4	5	1	2	3	4
1	B. pseudomallei K96243	+	+	+	+	+	+w	+w	–	+w
2	B. thailandensis E264	+	+	+	+	+	+w	+w	–	+w
3	B. mallei NCTC10248	+	+	+	+	+	+w	+w	–	+w
4	B. cepacia U668	+w	–	+	+w	+w	+w	+	+w	–
5	*B. multivorans* LMG16660	–	–	–	–	–	–	–	–	–
6	*B. oklahomensis* C7532	–	–	+	–	–	–	–	–	–
7	*B. vietnamiensis* LMG6999	+	+w	+	–	–	+	+w	+w	–
8	*B. ubonensis* DMST886	–	–	–	–	–	–	–	–	–
9	E. coli ATCC 25922	–	–	–	–	–	–	+w	–	–

a +, positive; +w, weakly positive; –, negative.

## DISCUSSION

This study demonstrated the different chemical structures and immunological characteristics of the LPSs isolated from seven *Burkholderia* species, including pathogenic and nonpathogenic species, and compared these with the chemical structures and immunological characteristics of B. pseudomallei LPS. Our results showed that eight genetically related *Burkholderia* species, including B. pseudomallei, contained penta- and tetra-acylated lipid A species modified with 4-amino-4-deoxy-arabinose (Ara4N). All had a bisphosphorylated glucosamine disaccharide backbone, which is a common component of Gram-negative bacterial lipid A (32). By use of MALDI-TOF MS, *Burkholderia* lipid A species can be divided into three groups based on the differences in ion masses at *m/z* 1,511, 1,642, 1,773, and 1,926 and the fatty acid component of lipid A (group I, consisting of B. pseudomallei, B. multivorans, and B. vietnamiensis; group II, consisting of B. thailandensis, B. mallei, B. cepacia, and B. ubonensis; and group III, consisting of B. oklahomensis only). Our data also demonstrated that LPS can be categorized into two distinct groupings based on SDS-PAGE profiles of the OPS: group I, comprising B. pseudomallei, B. mallei, and B. thailandensis, which showed similar repeating bands with uniform spacing (LPS type A ladder), and group II, comprising B. cepacia, B. multivorans, B. oklahomensis, B. vietnamiensis, and B. ubonensis, which demonstrated LPS ladders different from those of group I and from each other. Even though three lipid A groups were observed by MALDI-TOF MS and two groups by SDS-PAGE analysis, the LPSs from all eight *Burkholderia* species were able to induce strong TLR4-dependent NF-κB activation at LPS concentrations of ≥10 ng/ml. However, some differences in TLR4-dependent NF-κB activation unrelated to these LPS groups were detected when cells were stimulated at LPS concentrations of 0.1 and 1 ng/ml. Furthermore, immunoblot analysis revealed various degrees of cross-reactivity of these LPSs against sera from melioidosis patients, as well as reactions with sera from healthy donors.

We demonstrated previously that the lipid A moieties of B. pseudomallei are conserved among different clinical B. pseudomallei isolates (24). The lipid A structures of B. pseudomallei, B. thailandensis, and B. mallei and the members of the Bcc (B. cepacia, B. multivorans, and B. vietnamiensis) have been described previously as penta- and/or tetra-acylated and modified with Ara4N; these have been shown to be potent activators of human TLR4 (7, 24–29). Others have shown that the lipid A structure of C_14:0_(2-OH) fatty acid substituted to the lipid A backbone was unique to B. pseudomallei; it was not found in B. thailandensis (26). Our results confirm that lipid A species acylated with C_14:0_(2-OH) fatty acid are present in B. pseudomallei but are not detected in B. thailandensis. The different immunological activities of B. pseudomallei and B. thailandensis may be due to the presence of C_14:0_(2-OH) fatty acid in the former (26).

Zughaier et al. reported that B. cepacia LPS is a more potent tumor necrosis factor alpha (TNF-α) stimulator than LPSs from Pseudomonas aeruginosa and Stenotrophomonas maltophilia in human MonoMac6 macrophages (33). Using a U937 monocytic cell line, McKeon et al. showed that B. multivorans LPS induces higher TNF-α levels by the JNK (Jun N-terminal protein kinase) mitogen-activated protein (MAP) kinase signaling pathway than the LPSs of P. aeruginosa and Staphylococcus aureus (34). Other studies demonstrated that the lipid A structures of Bcc species, including B. cepacia, B. multivorans, and B. vietnamiensis, and of B. mallei represent tetra- and penta-acylated GlcN disaccharide backbones possessing two C_14:0_(3-OH) residues in ester linkage and two C_16:0_(3-OH) residues in amide linkage, one of which is substituted by a secondary C_14:0_ residue at C-2 (25, 28–30). These reports potentially lead to confusion, because different methods and immune cell lines were used in each of these studies. A strength of our data is that we used a standardized method to compare the structures and biological activities of these *Burkholderia* species. In this study, we confirmed that the LPSs of all the different *Burkholderia* species, regardless of their virulence or lack of virulence, induced TLR4-dependent NF-κB responses on HEK-Blue hTLR4 cells. Compared with B. pseudomallei LPS, the LPSs of B. mallei, B. cepacia, B. oklahomensis, B. vietnamiensis, and B. ubonensis exhibited significantly lower levels of NF-κB activation. Factors that might induce the variation in the proinflammatory activities of LPS include primary structure, namely, fatty acids, polar heads, and carbohydrate components (18). The observation of lower TLR4-stimulatory response effects for the LPSs of other *Burkholderia* species than for B. pseudomallei LPS may result from different lipid A structures that remain undetected in MALDI-TOF MS profiles, from the carbohydrate components of LPS, such as O-polysaccharide, or from the core oligosaccharide. Lipid A variation in several pathogenic Gram-negative bacteria has been shown to be associated with different induction of innate immune responses. However, our study showed that TLR4-dependent NF-κB responses on HEK-Blue hTLR4 cells for eight different *Burkholderia* species did not correlate with the lipid A structures characterized by MALDI-TOF MS profiles. It is possible that other factors, such as the interference of O-polysaccharide with the binding of LPS to the TLR4–MD2 complex, also have effects on TLR4-dependent NF-κB activation (24).

In contrast to the lipid A analysis, our SDS-PAGE analysis demonstrated that *Burkholderia* species with similar lipid A spectra may express different LPS ladder patterns, potentially representing differences on OPS. In agreement with previous studies, this study demonstrated that the same LPS group (referred to as the type A ladder) was presented by B. pseudomallei, B. thailandensis, and B. mallei (23, 35); 97% of B. pseudomallei isolates from Thailand and 80% of isolates from Australia expressed LPS type A (22, 35). B. cepacia, B. multivorans, B. oklahomensis, B. vietnamiensis, and B. ubonensis showed LPS ladders different from each other, although some of them are members of the same lipid A group. Specifically, we showed previously that OPS is a potential diagnostic target antigen for melioidosis (36, 37). In this study, we found that LPSs from genetically closely related *Burkholderia* species reacted with sera from melioidosis patients with known infections by LPS type A. Sera from melioidosis patients cross-reacted with LPSs of closely related *Burkholderia* species, namely, B. thailandensis, B. mallei, B. cepacia, B. oklahomensis, and B. vietnamiensis, while sera from healthy donors in northeast Thailand reacted with LPSs from B. pseudomallei, B. thailandensis, B. mallei, B. cepacia, and B. vietnamiensis. This is in agreement with a previous study showing that a serum sample from an Australian melioidosis patient infected with B. pseudomallei (type A LPS) cross-reacted with the LPSs of B. thailandensis and B. oklahomensis strains with OPS biosynthesis genes similar to those of B. pseudomallei (23). The variation in the cross-reactivities of sera from individual melioidosis patients and healthy donors to these LPSs may result from the fact that individuals have been exposed to other *Burkholderia* species and have raised an LPS-specific immune response. These *Burkholderia* species may share some common O-polysaccharide structures. The cross-reactivity of melioidosis sera to LPSs from different *Burkholderia* species has important implications for the use of serological diagnostics based on the detection of antibody to OPS. Therefore, the species-specific subunit of the OPS molecule in *Burkholderia* species has the potential for further development and could lead to an improvement of the current serodiagnostic test. In future studies, it would be informative to examine sera from patients infected with other, closely related *Burkholderia* species for antibodies to the OPSs of Burkholderia pseudomallei and other *Burkholderia* species.

MALDI-TOF MS based on main spectral profile (MSP) and *recA* gene sequencing have been reported previously for the identification and grouping of *Burkholderia* species (38). A phylogenetic tree based on the MSP and *recA* genes of *Burkholderia* isolates divides the bacteria into two major branches. The first contains B. multivorans, B. vietnamiensis, B. ubonensis, and B. cepacia, and the second contains B. thailandensis, B. oklahomensis, B. mallei, and B. pseudomallei (38). The lipid A profiles that we generated by MALDI-TOF MS and GC did not arrange these isolates into the same groups, nor could we identify species-specific characteristics.

In conclusion, our study has demonstrated the variation of the lipid A structural features and immunological characteristics of the LPSs in *Burkholderia* species. Importantly, LPSs from different *Burkholderia* species can stimulate TLR4-dependent NF-κB activation, and immunoblot analysis demonstrated that serum samples from melioidosis patients and healthy donors reacted with OPSs of *Burkholderia* species other than B. pseudomallei. Further studies are required to characterize the lipid A structures and biological activities of more isolates for each species and to evaluate the role and full structure of *Burkholderia* species OPS.

## MATERIALS AND METHODS

### Ethics statement.

The study was approved for ethical review by the Ethics Committee of the Faculty of Tropical Medicine, Mahidol University, Bangkok, Thailand (approval number MUTM 2015-002-01), and Udon Thani Hospital, Udon Thani, Thailand (approval number 0032.102/034). Written informed consent was obtained from all subjects enrolled in this study.

### Bacterial strains and culture conditions.

All *Burkholderia* species were cultivated in a biosafety level 3 (BSL3) laboratory. Eight *Burkholderia* species comprising 19 strains were used in this study: B. pseudomallei strains K96243 and 1026b; B. thailandensis strains E264 and E555; B. mallei strains NCTC10248, ATCC 23344, EY2236, and EY2237; B. cepacia strains U668, 10223, 2.1B, 39628, and LNT40; B. multivorans LMG16660; B. oklahomensis C7532, C7533, and C6786; B. vietnamiensis LMG6999; and B. ubonensis DMST886. Unless stated otherwise, bacteria were cultured on Trypticase soy agar (TSA) and incubated at 37°C for 16 to 18 h. All isolates were stored in Trypticase soy broth (TSB) with 15% glycerol at –80°C.

### Serum specimens.

Serum samples from five culture-confirmed melioidosis patients who were admitted to Udon Thani Hospital, Udon Thani, northeast Thailand, between July 2015 and September 2017, and four serum samples obtained from healthy donors who resided in the same province, were used to detect antibody reactivity to the LPSs of different *Burkholderia* species. The serum samples were kept frozen at –80°C until use.

### Lipid A extraction and MALDI-TOF MS analysis.

Lipid A was extracted from bacteria by use of hot ammonium isobutyrate as described previously (24). One microliter of supernatant was spotted onto stainless steel target plates, allowed to dry in air, and overlaid with 1 μl of a 20-mg/ml norharmane matrix (Sigma-Aldrich, St. Louis, MO, USA). Each spot was measured in 500 shot steps for a total of 3,000 laser shots using a Bruker Autoflex Speed MALDI-TOF mass spectrometer (Bruker Daltonics, Bremen, Germany) operated in negative-ion reflectron mode. The mass spectra were acquired in the mass range of 1,500 to 2,000 to cover penta- and tetra-acylated lipid A species and were calibrated with an electrospray ionization (ESI) tuning mix (Agilent Technologies, Santa Clara, CA, USA) as described previously (24). The mass spectra were processed for smoothing and baseline subtraction using FlexAnalysis, version 3.4 (Bruker Daltonics, Bremen, Germany) (24).

### Purification of LPSs.

Lipopolysaccharides (LPSs) were extracted using a modified hot-water–phenol method and were further treated with RNase A, DNase I, and proteinase K to ensure the removal of contaminating nucleic acids and proteins as described previously (24). All LPS samples from the first round of phenol purification were resuspended at 1 to 2% (wt/vol) in chloroform-methanol at a 2:1 (vol/vol) ratio and were centrifuged at 10,000 rpm for 10 min, and extraction was repeated 3 more times. The pellet was suspended in endotoxin-free water and was lyophilized. This process was used to remove contaminating phospholipids as described by Folch et al. (39).

The lyophilized LPSs were further reextracted with phenol to remove contaminating endotoxin and proteins as described elsewhere (40). Briefly, 2.5 mg of lyophilized LPSs was reextracted with 500 μl of endotoxin-free water containing 0.2% trimethylamine (TEA) and 0.5% deoxycholate (DOC), followed by 500 μl of phenol, and was centrifuged at 10,000 × *g*. Then the top aqueous layer was transferred to a new tube, and the phenol phase was subjected to reextraction with 500 μl of 0.2% TEA and 0.5% DOC. The pooled aqueous phase was precipitated with 75% ethanol and 30 mM sodium acetate at –20°C for 1 h. The pellet was washed with 1 ml of 100% cold ethanol and was air dried. Finally, the purified LPSs were suspended in endotoxin-free water and were lyophilized.

Protein contamination was determined using Coomassie blue staining (41) and a bicinchoninic acid (BCA) assay (Pierce, Rockford, IL, USA). Coomassie blue staining of all purified LPSs showed no protein bands, and the BCA assay detected <1% contaminating protein.

### Fatty acid analysis.

Bacteria were cultured on TSA plates at 37°C for 16 to 18 h, harvested with a 10-μl loop, and suspended in 18 ml of phosphate-buffered saline (PBS). Bacteria were inactivated by adding phenol to obtain a 1% final concentration, and the bacterial suspension was incubated at 37°C overnight. The cell pellet was collected by centrifugation at 12,000 × *g* for 3 min and was washed twice with 20 ml of PBS. The pellet was resuspended in 5 ml PBS. Five hundred microliters of the bacterial suspension was plated on TSA for a sterility test. The remaining bacterial suspension was lyophilized and collected as a dried bacterial cell. LPS fatty acids were extracted from dried cells and were derivatized to fatty acid methyl esters prior to gas chromatography (GC) analysis as described previously (24, 42).

### Silver staining and immunoblot analysis.

LPS profiles and the immunoreactivities of serum antibodies to the LPSs of different *Burkholderia* species were analyzed using silver staining and Western blotting, respectively. LPSs were extracted using the proteinase K digestion method as described previously (43). SDS-PAGE was performed on a 12% gel according to the method described by Laemmli (44). The LPS profile on the gel was detected with a silver stain (43, 45). Antibody reactivity to these LPSs was demonstrated using the serum samples from melioidosis patients and healthy donors at a serum dilution of 1:1,000 and horseradish peroxidase (HRP)-conjugated rabbit anti-human IgG (dilution, 1:2,000; Dako A/S, Copenhagen, Denmark) as described previously (43). E. coli LPS was used as the control for immunoblot analysis.

### Cell culture and TLR4 activation.

TLR4-transfected human embryonic kidney cells (HEK-Blue hTLR4 cells; InvivoGen, San Diego, CA, USA) were cultured and maintained at 37°C under 5% CO_2_ in complete Dulbecco’s modified Eagle medium (DMEM) (Gibco, NY, USA) containing 10% heat-inactivated fetal bovine serum (FBS) and 1× HEK-Blue selection medium (InvivoGen, San Diego, CA, USA). The cells were seeded at 2.5 × 10^4^/well in a 96-well plate and were stimulated with purified LPSs at 0.1, 1, 10, 100, or 1,000 ng/ml for 24 h. The ultrapure TLR4 ligand of Escherichia coli O111:B4 LPS (Sigma-Aldrich, St. Louis, MO, USA) was used as a positive control. The activation of NF-κB in HEK-Blue hTLR4 cells in response to TLR4 agonists was determined by a SEAP (secreted embryonic alkaline phosphatase) reporter assay at a wavelength of 620 nm using a microplate reader (Tecan, Grödig, Austria) (24).

### Statistical analysis.

Statistical analysis was performed using GraphPad Prism (version 6; GraphPad Software, La Jolla, CA, USA). One-way analysis of variance (ANOVA) was used to test the differences in mean levels of NF-κB activation by the LPSs of different *Burkholderia* species and to compare those levels with that for B. pseudomallei LPS. The data are presented as means ± standard deviations (SD). Differences were considered statistically significant at a *P* value of <0.05.
